# Association of preoperative frailty with the risk of postoperative delirium in older patients undergoing hip fracture surgery: a prospective cohort study

**DOI:** 10.1007/s40520-023-02692-5

**Published:** 2024-01-31

**Authors:** Chunyu Feng, Haotian Wu, Ziheng Qi, Yuzhi Wei, Bo Yang, Haolin Yin, Siyi Yan, Lu Wang, Yangyang Yu, Juanjuan Xie, Xueyan Xing, Shumin Tu, Huan Zhang

**Affiliations:** 1 School of Clinical Medicine, Tsinghua University, Beijing, China; 2 Department of Anesthesiology, Beijing Tsinghua Changgung Hospital, No.168 Litang Road, Changping District, Beijing, 102218 China; 3 Department of Plastic Surgery, Beijing Tsinghua Changgung Hospital, No.168 Litang Road, Changping District, Beijing, 102218 China; 4 Department of Orthopedic, Beijing Tsinghua Changgung Hospital, No.168 Litang Road, Changping District, Beijing, 102218 China

**Keywords:** Frailty, Elderly, Postoperative delirium, Hip fracture surgery, Perioperative neurocognitive disorders

## Abstract

**Objective:**

This study aimed to explore the correlation between preoperative frailty and the risk of postoperative delirium (POD) in older patients undergoing hip fracture surgery.

**Methods:**

In total, 148 patients with hip fractures who were admitted to Tsinghua Changgung Hospital (Beijing, China) between January 2022 and January 2023 were involved in this study. Preoperative frailty scales were assessed, of which the CAM scale was postoperatively administered every morning and evening on days 1, 2, 3, 5, and 7. Binary logistic regression analysis was conducted to determine the correlation between preoperative frailty and the risk of POD.

**Results:**

Among 148 older patients with hip fractures, 71 (48.0%) were identified as preoperative frail and 77 (52.0%) as non-frail. The overall incidence of POD on day 7 was 24.3% (36/148), and preoperative frailty was associated with a significantly higher risk of POD compared with non-frailty (42.3% vs. 7.8%, *P* < 0.001). The binary logistic regression analysis revealed that preoperative frailty was noted as an independent risk factor for the risk of POD in older patients undergoing hip fracture surgery (*P* = 0.002).

**Conclusion:**

Preoperative frailty increased the risk of POD in older patients undergoing hip fracture surgery.

**Discussion:**

Preoperative assessment of frailty in geriatric hip surgery can timely identify potential risks and provide interventions targeting frailty factors to reduce the incidence of POD in older patients undergoing hip fracture surgery. The findings suggested that preoperative frailty could increase the risk of POD in older patients undergoing hip fracture surgery. Further research is necessary to determine whether perioperative interventions aimed at enhancing frailty can mitigate the risk of POD and improve prognosis in older patients undergoing hip fracture surgery.

## Introduction

Frailty is a non-specific condition characterized by a decline in physiological reserves among the elderly, resulting in increased vulnerability and reduced resistance to stressors. Numerous studies have demonstrated that frail patients are susceptible to negative clinical outcomes, such as falls, prolonged hospital stay, and heightened postoperative mortality even in response to minor external stimuli [1–4]. The prevalence of frailty has been reported to range from 18.6% to 56% [5], and frailty is particularly prevalent among hip fracture patients [6–9].

Hip fracture represents a noticeable public health challenge, given the anticipated rise in hip fracture incidence as the global population ages. The International Osteoporosis Foundation reported that there were 1.6 million hip fracture patients worldwide in 2000, with projections indicating an increase to between 4.5 and 6.3 million by 2050 [10, 11]. According to the latest hip fracture projections for Asia, the number of hip fracture patients will increase from 11.2 million in 2018 to 2.56 million in 2050, a 2.28-fold increase, and the direct cost of hip fractures will increase from $9.5 billion in 2018 to $15 billion in 2050, indicating a 1.59-fold increase [12]. Up to 20–24% of patients with hip fractures have been reported to experience one-year mortality rates, and the risk of death may persist for more than five years [13]. Older patients with hip fractures mainly suffer from multiple comorbidities, resulting in a perioperative mortality rate that is more than 10% higher due to several complications [14–16]. Postoperative delirium (POD) is a prevalent complication in older patients with hip fractures undergoing surgery, with reported prevalence rates ranging from 28 to 61% [17].

Notably, POD, an acute attention and cognitive disorder, is a prevalent, severe, expensive, and often fatal condition in older adults. It can result in elevated risks of falls, hospitalization, readmission, and mortality rates [18–21]. The incidence of POD in older patients ranges from 5 to 50%, and it is widely accepted that the etiology of POD is multifactorial [18, 22, 23].

It has been demonstrated that frailty is a predisposing factor for postoperative complications in patients with hip fractures [1, 24–26]. It was evidenced that a higher level of frailty may be associated with the development of delirium [27, 28]. Conversely, few domestic and international studies have concentrated on the correlation between frailty and the risk of POD in older patients undergoing hip fracture surgery. In the present study, 148 older patients who underwent hip fracture surgery were enrolled to investigate the association between preoperative frailty and the risk of POD in older patients undergoing hip fracture surgery, providing clinical guidance.

## Materials and methods

### Study design

This is a prospective observational study that recruited patients aged 60 years and older who underwent hip surgery in the Tsinghua Chang Gung Hospital (Beijing, China). The study protocol (Appendix A) was approved by the Ethics Committee of the Tsinghua Chang Gung Hospital (Approval No. 21277-0-01) and was registered on ClinicalTrials.gov (identifier: NCT05246254). Written informed consent was obtained from all eligible patients prior to commencing the study.

### Inclusion and exclusion criteria

A total of 148 patients who were hospitalized between January 1, 2022, and January 31, 2023, were included in the study. The inclusion criteria were as follows: Participants who aged 60 years or above; The presence of hip fractures; Participants who signed the informed consent form; Participants with American Society of Anesthesiologists (ASA) class I-IV; Procedures must be administered by the same anesthesia and surgical team. The exclusion criteria were as follows: Participants who were incapable of providing informed consent or those who declined their participation; Participants with a history of cognitive impairment; Participants who were unable to cooperate in completing the cognitive function test; Patients experiencing mental confusion during the initial assessment; Participants who were diagnosed with mental illnesses or substance use disorders; Participants with incomplete or missing data at follow-up.

### Grouping

Participants were categorized into two groups based on their frailty index (FI) scores, including the non-frail group (FI < 0.25) and the frail group (FI ≥ 0.25).

### Standardized anesthetic management

(i) The temperature of operating room was maintained within the range of 20–23 °C, while humidity was controlled at 50%-60%; (ii) Active measures were taken to keep the room warm via combination of warming blankets and fluid warming techniques; (iii) Upon entering the operating room, an 18G IV catheter was inserted into the left forearm to establish an IV line, and lactated Ringer’s solution was infused at a rate of 1 ml.min^−1^ to maintain venous patency. Routine monitoring was continuously conducted, which included electrocardiography, non-invasive blood pressure measurement, and pulse oximetry. Systolic blood pressure (SBP), heart rate (HR), and blood oxygen saturation were evaluated every 3 min. The combined spinal-epidural (CSE) puncture was routinely performed at the L3-4 levels by an experienced anesthesiologist while the patient was in the right lateral decubitus position. A 16-gauge Tuohy needle was utilized to perform epidural puncture using a paramedian approach. Following identification of the entry into the epidural space, a 25-gauge Whitacre needle was introduced through the epidural needle. Once the cerebrospinal fluid was detected, an isobaric dose of 0.75% ropivacaine was administered via the Whitacre needle. After administering ropivacaine, an epidural catheter was advanced 3 cm through the Tuohy needle into the epidural space. The patient was then placed in the supine position with left uterine displacement achieved by inserting a wedge under the right hip. (iv) Adjunctive sedation was provided via continuous infusion of low-dose dexmedetomidine (0.1–0.3 μg.kg^−1^.h^−1^). (v) Invasive arterial blood pressure monitoring is routinely employed in patients with severe cardiopulmonary comorbidities or compromised general condition; (vi) It is recommended to refine goal-directed hemodynamic management and blood volume optimization measures to maintain optimal hemodynamic status and sustain adequate blood pressure; (vii) It is essential to ensure that the Hb level remains at no less than 90 g/L.

### Observational indices

(i) Preoperative factors, including age, gender, body mass index (BMI), ASA classification, smoking history, frailty status, history of heart disease, hypertension and stroke, as well as malnutrition (albumin < 34 g/L); (ii) Surgery and anesthesia data encompass operation time and intraoperative blood loss; (iii) Postoperative variables, such as confusion assessment method (CAM) scale scores, length of stay in intensive care unit (ICU), and duration of hospitalization.

### Assessment criteria

#### Diagnosis of delirium

The CAM scale is the most widely used tool for delirium screening worldwide [29, 30]. It has a sensitivity of 95–100% and a specificity of 90–95%. The measure consists of a rapid, standard assessment of the main symptoms of delirium and its typical course. The corollary symptoms of CAM are acute exacerbation and/or fluctuating course and attention deficit disorder. Delirium was evaluated postoperatively on days 1, 2, 3, 5, and 7 by a highly trained senior anesthesiologist with rigorous neurology training using the CAM scale.

#### Assessment of frailty

The FI is a cumulative deficit model that was developed by Rockwood and Mitnitski. This model is based on the theory of health deficits [31]. The FI has noticeable applications in reflecting health functional status and changes, health service needs, public health management, and interventions. It has exhibited the strongest relationship with mortality and comorbidity compared with other frailty scales [32, 33]. The FI is defined as the ratio of an individual’s potential unhealthy measures to all measures at a specific point in time. The variables considered for this index encompass physical, functional, psychological, and social health aspects. These variables are selected based on certain principles, such as being acquired, age-related, having biologically plausible adverse health consequences, and not being prematurely saturated. In this study, a total of 40 deficit variables were included[33–35]. It is widely acknowledged that a FI of ≥ 0.25 indicates frailty in the elderly, while an FI < 0.25 denotes non-frailty among this population [36]. The FI is a reliable tool for assessing the degree of frailty and predicting clinical outcomes in older adults, and it has gained widespread attention in both clinical research and community settings[37, 38]. Trained researchers perform the frailty assessment independently from daily delirium assessments.

### Outcome indicators

The primary outcome was the incidence of POD in older patients with hip fractures. The secondary outcomes included operation time, intraoperative blood loss, length of hospital stay (defined as the time from admission to discharge), and length of stay in the ICU.

### Statistical analysis

Descriptive statistics were generated for the analytical cohort and stratified by the frailty status (developed vs. not developed). The distribution of continuous variables was assessed by Kolmogorov–Smirnov test. Normally distributed variable (e.g., BMI) was presented as mean and standard deviation (SD), while abnormally distributed variables (e.g., age, FI, operation time, intraoperative blood loss, length of hospital stay, and length of stay in ICU) were characterized as the median and interquartile range (IQR). Categorical variables were reported as count and percentage. The statistical significance between groups was assessed using the *t*-test or the Wilcoxon rank-sum test for continuous variables, and the Chi-square test or the Fisher’s exact test for categorical variables. A binary logistic regression model was employed to assess the association between preoperative frailty and the risk of POD, in which POD and preoperative frailty were considered as dependent and independent variables, respectively. It was further attempted to adjust age, smoking status, heart disease history, stroke history, hypertension history, operation time, length of hospital stay, and length of stay in ICU in the logistic regression model, which were distributed differently between non-frail and frail groups through the univariate analysis. The association estimates were presented as odds ratios (ORs) with corresponding 95% confidence intervals (CIs). The statistical analysis was performed using SPSS 25.0 software (IBM, Armonk, NY, USA). A two-tailed *P* < 0.05 was considered statistically significant.

## Results

### Descriptive statistics

**Fig. 1:**
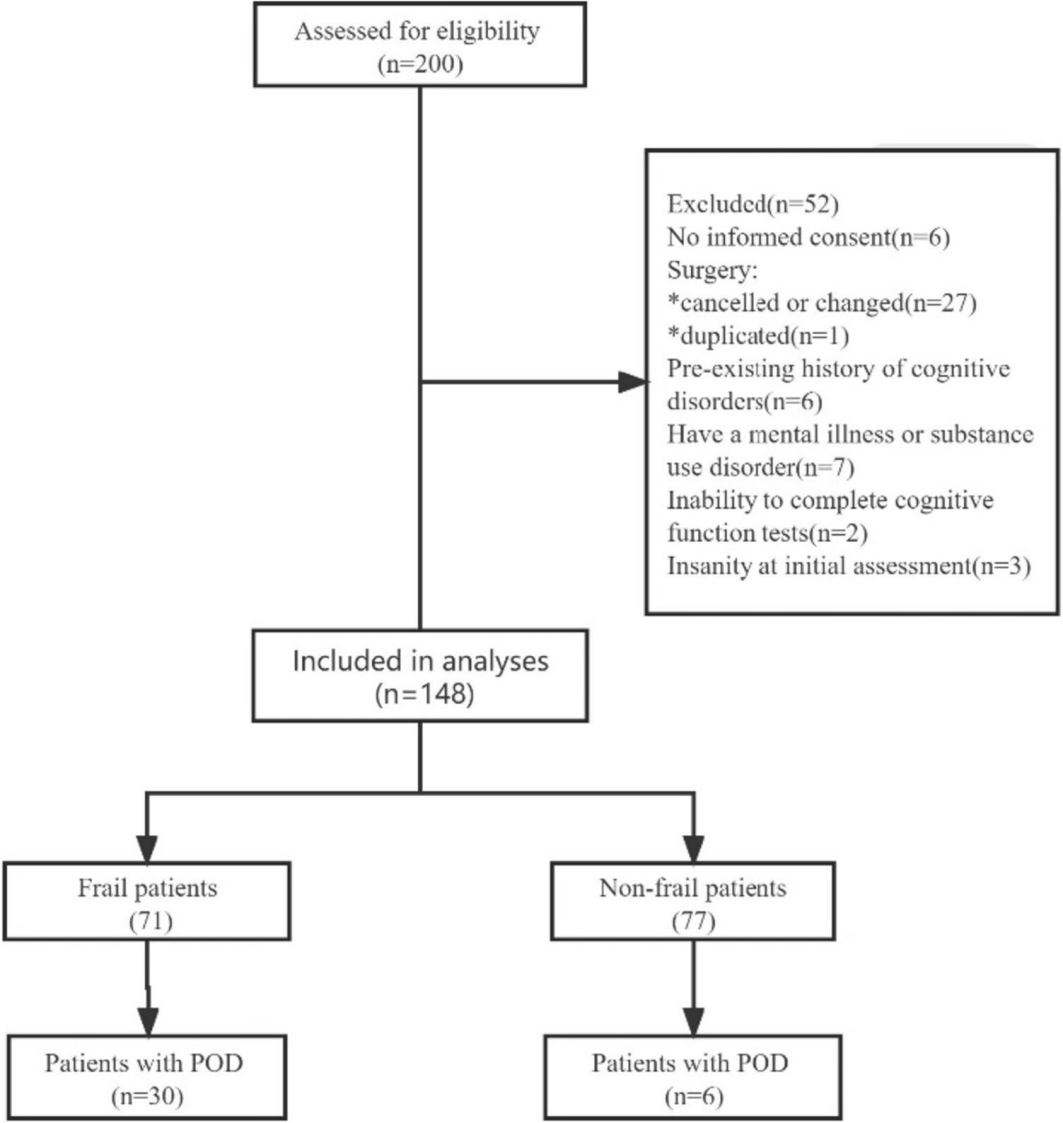
Depicts the flowchart of patient enrollment, with POD denoting postoperative delirium.

Between January 2022 and January 2023, a total of 200 patients underwent screening, of whom 148 patients were ultimately involved in the analysis (Fig. 1). No patient was lost to follow-up during the study period, and complete data were available for both primary and secondary outcomes.

The sociodemographic and clinical characteristics of the study population are presented in Table 1. Patients’ median age was 81.3 (IQR: 73.8, 83.7) years, and there were 41 (27.7%) male patients. The average BMI was 24.1 ± 4.6 kg/m^2^, the median operation time was 2.0 (IQR: 1.5, 2.5) hours, the median blood loss was 100.0 (IQR: 50.0, 200.0) mL, the median length of hospital stay was 11.0 (IQR: 8.0,14.8) days and the median length of stay in ICU was 0.0 (IQR: 0.0, 21.0) hours. Of these, 7 (4.7%) patients smoked, 55 (37.2%) patients had a history of heart disease, 50 (33.8%) patients had a history of stroke, 96 (64.9%) patients had a history of hypertension, and 20 (13.5%) patients were malnutrition.

**Table 1:** Main characteristics of patients according to their frailty index (FI) scores at hospital admission (*N* = 148)

Variable	Total *N* = 148	Non-frail group (FI < 0.25) *N* = 77	Frail group (FI ≥ 0.25) *N* = 71	*P*
Demographic characteristic				
Age*, median (IQR), years	81.3 (73.8, 86.7)	77.3 (69.5, 84.3)	83.4 (75.8, 88.4)	0.001
Gender, *N* (%)				0.177
Male	41.0 (27.7)	25.0 (32.5)	16.0 (22.5)	
Female	107.3 (72.3)	52.0 (67.5)	55.0 (77.5)	
Health-related characteristics				
Smoking*, *N* (%)	7 (4.7)	4 (5.2)	3 (4.2)	1.000
Alcohol drinking, *N* (%)	4 (2.7)	3 (3.9)	1 (1.4)	0.621
Health-related characteristic				
FI*, median (IQR)	0.23 (0.13, 0.34)	0.13 (0.08, 0.16)	0.35 (0.30, 0.40)	< 0.001
BMI, mean ± SD, kg/m^2^	24.1 ± 4.6	24.0 ± 3.9	24.2 ± 5.4	0.769
Heart disease*, *N* (%)	55 (37.2)	22 (28.6)	33 (46.5)	0.024
Stroke*, *N* (%)	6 (4.1)	0 (0.0)	6 (8.5)	0.011
Hypertension*, *N* (%)	96 (64.9)	41 (53.2)	55 (77.5)	0.002
Malnutrition, *N* (%)	20 (13.5)	7 (9.1)	13 (18.3)	0.148
ASA classification*, *N* (%)				< 0.001
Class I	0 (0.0)	0 (0.0)	0 (0.0)	
Class II	15 (10.1)	15 (19.5)	0 (0.0)	
Class III	123 (83.1)	59 (76.6)	64 (90.1)	
Class IV	10 (6.8)	3 (3.9)	7 (9.9)	
Primary outcome				
Postoperative delirium, *N* (%)	36.0 (24.3)	6.0 (7.8)	30 (42.3)	< 0.001
Secondary outcomes				
Operation time*, median (IQR), hours	2.0 (1.5, 2.5)	2.0 (1.5, 2.5)	2.0 (1.5, 2.0)	0.010
Intraoperative blood loss, median (IQR), mL	100.0 (50.0, 200.0)	100.0 (50.0, 200.0)	100.0 (50.0, 200.0)	0.507
Length of hospital stay*, median (IQR), days	11.0 (8.0,14.8)	10.0 (8.0, 12.0)	12.0 (9.0, 16.0)	< 0.001
Length of stay in ICU*, median (IQR), hours	0.0 (0.0, 21.0)	0.0 (0.0, 11.0)	17.5 (0.0, 24.0)	< 0.001

*ASA* American Society of Anesthesiologists, *BMI* Body mass index, *FI* Frailty index, *SD* Standard deviation

Differences between non-frail group and frail group in age, FI, operation time, intraoperative blood loss, length of hospital stay, and length of stay in ICU were compared by the Wilcoxon rank-sum test. Differences between non-frail group and frail group in BMI were compared by *t* test. Differences between non-frail group and frail group in gender, heart disease, hypertension, and postoperative delirium were compared by the Chi-square test. Differences between non-frail group and frail group in smoking, alcohol drinking, stroke, malnutrition, and ASA classification were compared by the Fishers’ exact test

*Differences with statistical significance (*P* < 0.05) between non-frail group and frail group

### Comparison of perioperative indicators between the frail and non-frail groups

Upon admission, 77 (52.0%) patients were categorized as non-frail with FI scores < 0.25, while the remaining 71 (48.0%) patients were classified as frail due to their higher FI scores. There were statistically significant differences in age, smoking status, history of heart disease, history of stroke, history of hypertension, operation time, length of hospital stay, length of stay in ICU, and the presence of delirious (*P* < 0.05). Although no significant differences were found in operation time and intraoperative bleeding between the two groups, those who were frail had longer ICU stays and hospitalizations (Table 1). After undergoing surgical intervention, preoperative frail patients were found to have a significantly higher incidence of POD compared with non-frail patients. Specifically, 30 POD cases were identified in the frail group (42.3% 30/71), while only 6 cases were found in the non-frail group (7.8%, 6/77).

### Binary logistic regression analysis of risk factors for the occurrence of POD

Binary logistic regression analysis of risk factors influencing the occurrence of POD was carried out. By utilizing a binary logistic regression model (Table 2), association estimates were obtained between frailty and delirium. The findings indicated that frailty alone was significantly associated with the risk of POD (OR = 5.169, 95% CI 1.795–14.890, *P* = 0.002).

**Table 2:** Binary logistic regression models for variables associated with the risk of postoperative delirium

Variable	*β*-value	*β*-value standard error	Wald Chi-squared value	*P*	OR (95% CI)
Frail	1.643	0.540	9.262	0.002	5.169 (1.795, 14.890)
Age	0.061	0.031	4.002	0.045	1.063 (1.001, 1.128)
Smoking	0.615	1.078	0.325	0.568	1.850 (0.223, 15.310)
Heart disease	− 0.354	0.501	0.498	0.480	0.702 (0.263, 1.875)
Stroke	1.309	0.932	1.971	0.160	3.701 (0.595, 23.002)
Hypertension	0.306	0.547	0.312	0.576	1.357 (0.465, 3.963)
Operation time	− 0.066	0.296	0.050	0.823	0.936 (0.524, 1.672)
Length of hospital stay	0.033	0.037	0.811	0.368	1.034 (0.961, 1.112)
Length of stay in ICU, hours	0.011	0.007	2.164	0.141	1.011 (0.996, 1.026)

*OR* odds ratio, *95% CI* 95% confidence interval

## Discussion

In this study, the relationship between preoperative frailty and the risk of POD in older patients with hip fractures was investigated, and it was revealed that preoperative frailty is a significant predictor for the increased risk of POD. The findings were consistent with those of previous experimental studies examining the association between preoperative frailty and the risk of POD in older adults [39–45]. Furthermore, the present study contributes to the existing literature on strategies for preventing delirium in older patients with hip fractures by assessing the relationship between preoperative frailty and POD. These results provide further evidence, supporting efforts to reduce the incidence of POD in this vulnerable population.

In hospitalized elderly patients, the presence of frailty is associated with a higher incidence of postoperative complications, including POD. A pilot study conducted by Leung et al. with a sample size of 63 demonstrated that preoperative frailty was prevalent among older patients undergoing noncardiac surgery and independently associated with the development of POD. The mentioned study utilized CAM and reported an incidence rate of 25% for POD in the cohort [46].

In a retrospective study of 556 hospitalized elderly orthopedic trauma patients, Shooka Esmaeeli et al. found that preoperative frailty significantly increased the risk of POD. Researchers utilized the CAM to assess the occurrence of POD and observed its presence in 14% of patients [47]. Similarly, in a prospective study involving 383 patients undergoing total joint arthroplasty, Yun Chen et al. reported an incidence rate of 17.2% for POD, and frailty was identified as an independent predictor of its development [44]. A recent meta-analysis comprising 15 cohort studies involving 3250 adult patients who underwent surgery revealed a preoperative frailty prevalence of 27.1% and a POD incidence of 15.8%. The analysis demonstrated that frailty was significantly associated with an increased risk of POD [48].

The existing effective strategies for preventing POD are generally multicomponent interventions, which may include the utilization of antipsychotics, dual-frequency index-guided anesthesia, and dexmedetomidine treatment [49].

Given the detrimental impact of POD on patient prognosis, there is a need to identify more effective interventions. According to the results of the present study, frailty was found to be independently associated with the risk of POD in older patients with hip fractures. Establishing a predictive model of frailty can identify patients who are at risk of POD and assist development of interventional strategies. This may reduce the incidence of POD in older patients with hip fractures, thereby decreasing medical costs and improving clinical prognosis.

Although the findings of the present study are intriguing, it is crucial to address its limitations. Firstly, although a prospective analysis was conducted that partially demonstrated the causal relationship between preoperative frailty and the risk of POD in older patients undergoing hip fracture surgery, the presence of multiple covariates could not be completely ruled out despite the efforts dedicated to adjusting for them, which might lead to residual confounding, affecting the results.

Secondly, this study was conducted solely at a single national tertiary academic medical center in an urban setting and exclusively on older patients who underwent hip fracture surgery. Therefore, the generalizability of the findings to other patient populations or practice sites might be limited. Thirdly, the FI was employed to categorize patients according to their level of frailty.

Although the FI is currently the most precise and convenient clinical tool for distinguishing frailty, it may not be feasible to determine the impact of varying degrees of frailty on the incidence of POD in older patients with hip fractures as this study only dichotomized the population into frail or non-frail groups.

Fourthly, CAM was employed as a surrogate marker for POD. Despite the high sensitivity and specificity of CAM in aggregating patient populations[50], some studies have suggested its suboptimal performance in patients undergoing surgery [51]. Therefore, there might be a likelihood of an elevated incidence of POD in this study cohort.

Finally, there is no universally accepted definition for the optimal time frame to define POD. To avoid overlooking delirious events that may occur postoperatively, POD was defined as a new onset of delirium within 7 days after surgery. However, due to the extended duration of the statistical analysis, there might be confounding factors unrelated to perioperative management.

In conclusion, the findings of this study suggest that preoperative frailty is a potentially modifiable risk factor for the risk of POD in older patients undergoing hip fracture surgery. Further large-scale multicenter studies are required to validate the findings and to further refine clinical decision-making utilizing frailty scores for stratifying frailty. This will enable scholars to determine whether perioperative interventions aimed at improving frailty can effectively reduce the risk of POD and enhance medical outcomes in this rapidly expanding patient population.

## Data Availability

The corresponding author can provide the raw data of this study upon reasonable request.
